# Modelling the current distribution and predicted spread of the flea species *Ctenocephalides felis* infesting outdoor dogs in Spain

**DOI:** 10.1186/s13071-017-2357-4

**Published:** 2017-09-19

**Authors:** Rosa Gálvez, Vicenzo Musella, Miguel A. Descalzo, Ana Montoya, Rocío Checa, Valentina Marino, Oihane Martín, Giuseppe Cringoli, Laura Rinaldi, Guadalupe Miró

**Affiliations:** 1 Departamento de Sanidad Animal, Facultad de Veterinaria, Universidad Complutense de Madrid, Avda. Puerta de Hierro s/n, Madrid, Spain; 2 Department of Health Sciences, Magna Græcia University, Catanzaro, Italy; 3 Unidad de Investigación, Academia Española de Dermatología y Venereología, Calle Ferraz, 100, Madrid, Spain; 4 Department of Veterinary Medicine and Animal Production, University of Naples Federico II, Regional Centre for Monitoring of Parasitic Diseases, Campania Region, Naples, Italy

**Keywords:** Fleas, *Ctenocephalides felis*, Dog, Climate change, GIS, Spain

## Abstract

**Background:**

The cat flea, *Ctenocephalides felis*, is the most prevalent flea species detected on dogs and cats in Europe and other world regions. The status of flea infestation today is an evident public health concern because of their cosmopolitan distribution and the flea-borne diseases transmission. This study determines the spatial distribution of the cat flea *C. felis* infesting dogs in Spain. Using geospatial tools, models were constructed based on entomological data collected from dogs during the period 2013–2015. Bioclimatic zones, covering broad climate and vegetation ranges, were surveyed in relation to their size.

**Results:**

The models builded were obtained by negative binomial regression of several environmental variables to show impacts on *C. felis* infestation prevalence: land cover, bioclimatic zone, mean summer and autumn temperature, mean summer rainfall, distance to urban settlement and normalized difference vegetation index. In the face of climate change, we also simulated the future distributions of *C. felis* for the global climate model (GCM) “GFDL-CM3” and for the representative concentration pathway RCP45, which predicts their spread in the country.

**Conclusions:**

Predictive models for current climate conditions indicated the widespread distribution of *C. felis* throughout Spain, mainly across the central northernmost zone of the mainland. Under predicted conditions of climate change, the risk of spread was slightly greater, especially in the north and central peninsula, than for the current situation. The data provided will be useful for local veterinarians to design effective strategies against flea infestation and the pathogens transmitted by these arthropods.

## Background

The cat flea, *Ctenocephalides felis*, is the most prevalent flea species detected on dogs and cats in Europe and other world regions [1–4]. The cosmopolitan distribution of *C. felis* and its tolerance to a broad range of environmental conditions ensure its success and survival [5, 6]. Recently, a need for flea control has been identified because of their worldwide distribution and transmission of flea-borne diseases, as well as flea allergy dermatitis (FAD) affecting companion animals [7, 8]. Most flea-borne pathogens are bacteria and some of them (e.g. *Bartonella* spp. and *Rickettsia* spp.) may cause important zoonoses [9]. *Ctenocephalides felis* can serve as the intermediate host for the tapeworm *Dipylidium caninum* and the filarial parasite *Acanthocheilonema reconditum*, both of which can parasitize humans [10, 11]. However, FAD remains as the major side effect of *C. felis* infestations in both dogs and cats. Flea saliva allergens on susceptible animals cause this allergic dermatitis, which is characterized by the presence of numerous papules and scabs on the back and around the neck [12].

Entomological surveillance and vector species occurrence data are essential to generate distribution models for arthropod vectors. This type of information is readily available from several web sites, such as FleaTickRisk (http://www.fleatickrisk.com) and VectorMap (http://vectormap.si.edu) [13, 14]. With the introduction of geographic information systems (GIS) and other geospatial tools, there is now growing interest in modelling vector distributions based on climate and environmental drivers for epidemiology studies and follow-up of arthropod vectors [15–17]. Statistical models help to determine the relative contribution of drivers to map vector occurrence or predict future vector distributions based on expected climate change [18]. In East Africa, scientists have had ample experience in surveying the bacterium *Yersinia pestis*, the causative agent of the plague, through GIS and Remote Sensing (RS) procedures [19–23]. However, so far only Beugnet et al. [14] have modelled the distribution of the cat flea affecting pets based on climate forecasts for a large geographical area [14].

In Spain, geo-environmental models have been recently established and validated for canine dirofilariosis (a mosquito-borne disease) [24, 25]. Also in Spain, vector occurrences and their future projections have been modelled for three tick species (*Dermacentor marginatus*, *Rhipicephalus turanicus* and *Hyalomma marginatum*) [26]; the bluetongue vector *Culicoides imicola* [27]; and the vectors of canine leishmaniosis, *Phlebotomus perniciosus* and *Phlebotomus ariasi* [28].

Flea distribution patterns may be more related to habitat than to host, since these insects are not very host-specific. Different geographical areas show a different spectrum of flea species. Predicting vector occurrence in specific regions will provide useful information on which to base the design of appropriate and focused control interventions [2, 29]. Hence, knowledge of the factors affecting flea species distributions in a given region is essential for the design of effective control protocols.

As part of our ongoing research, we recently described the spatial and temporal distributions of three flea species of the family Pulicidae infesting dogs in Spain [4]. As *C. felis* emerged as the most frequently detected and widely distributed throughout Spain, in the present study we used these data to map and model the current distribution and predicted spread of this flea species according to environmental variables and expected climate change.

## Methods

### Study design, dog sampling and entomological procedures

The study area, mainland Spain and the Balearic and Canary Islands, has been described in detail in Gálvez et al. [4]. A non-random sample of 1084 dogs was examined from late May 2013 to mid July 2015 for flea infestation at 42 sites covering six of the fourteen bioclimatic zones [30] listed in Table 1. The number of dogs surveyed in each bioclimatic belt was proportional to the surface area of each zone (Table 2), as explained in Gálvez et al. [4]. The sites surveyed covered a wide latitudinal and longitudinal range of the country, from south (Cádiz, 36°) to north (Lugo, 43°), and from west (Lugo, -7°) to east (Ibiza, 1°). The two sites sampled on the Canary Islands were the westernmost (Tenerife, -16°) and southernmost points surveyed (Gran Canaria, 27°).

**Table 1:** The bioclimatic zones of Spain and their altitude (minimum, maximum and mean)

Bioclimatic zones of Spain	Surface area (ha)	Altitude (metres above sea level)		
		Minimum	Maximum	Mean
A (Alpine)	74,972	1490	3153	2396
B (Subalpine)	426,884	739	3149	1882
C (Temperate oceanic)	3,788,382	112	2668	967
D (Temperate hyperoceanic)	3,018,127	0	1815	333
E (Cryoromediterranean)	9059	1792	3450	2548
F (Oromediterranean)	456,908	1008	3296	1757
G (Supramediterranean)	14,595,852	88	2427	961
H (Mesomediterranean)	21,239,216	0	1860	560
I (Thermomediterranean)	4,170,835	0	1391	173
K (Oromacaronesic)	1016	2721	3656	3177
L (Supramacaronesic)	21,756	1562	3149	2223
M (Mesomacaronesic)	111,752	127	2474	1275
N (Thermomacaronesic)	273,008	0	1637	560
O (Inframacaronesic)	326,940	0	1000	153

**Table 2:** Collection sites (*n* = 42) and *C. felis* infestation prevalences (dependent variable) by bioclimatic zone

Collection site	Locality/ Province	Bioclimatic zone	Latitude	Longitude	Dogs		Prevalence (%)
					*n*	Positive	
1	Guitiriz/ Lugo	Temperate oceanic (*n* = 93)	43.18	-7.89	35	7	11.4
2	Lugo/ Lugo		43.05	-7.53	50	0	0
3	Laracha/ A Coruña		43.20	-8.55	8	4	50.0
4	Artziniega 1/ Alava	Temperate hyperoceanic (*n* = 67)	43.14	-3.11	3	0	0
5	Artziniega 2/ Alava		43.12	-3.12	6	4	66.7
6	Izoria/ Alava		43.06	-3.03	10	10	70.0
7	Madaria/ Alava		43.04	-3.09	5	5	80.0
8	Maroño/ Alava		43.05	-3.06	6	3	0
9	Menagarai/ Alava		43.09	-3.08	5	4	80.0
10	Menoio/ Alava		43.07	-3.07	10	9	70.0
11	Respaldiza 1/ Alava		43.08	-3.09	4	0	0
12	Respaldiza 2/ Alava		43.08	-3.04	3	0	0
13	Respaldiza 3/Alava		43.08	-3.04	5	4	80.0
14	Respaldiza 4/ Alava		43.08	-3.05	1	1	100
15	Sojo/ Alava		43.09	-3.13	4	4	100
16	Soxoguti/ Alava		43.11	-3.12	5	3	60.0
17	El Casar 1/ Guadalajara	Supramediterranean (*n* = 164)	40.69	-3.43	12	6	50.0
18	El Casar 2/ Guadalajara		40.70	-3.43	21	21	100
19	Codos/ Zaragoza		41.30	-1.39	25	6	0
20	Alcarras/ Lleida		41.58	-0.49	38	7	5.3
21	Allariz/ Orense		42.22	-7.81	51	23	17.6
22	Navalcarnero/ Madrid		40.29	-4.00	17	11	58.8
23	Cañamero/ Caceres	Mesomediterranean (*n* = 496)	39.38	-5.40	50	21	42.0
24	Madrigal de la Vera/ Caceres		40.17	-5.41	41	2	0
25	Espinosa de Henares/ Guadalajara		40.89	-3.08	35	0	0
26	Achivel/ Murcia		38.07	-2.00	14	5	21.4
27	Morataya/ Murcia		38.21	-1.80	32	26	71.9
28	Utiel/ Valencia		39.59	-1.23	41	8	17.1
29	Puertollano/ Ciudad Real		38.67	-4.08	53	53	100
30	Arnedo 1/ La Rioja		42.22	-2.11	7	0	0
31	Arnedo 2/ La Rioja		42.22	-2.10	19	11	0
32	Herce/ La Rioja		42.22	-2.12	18	0	0
33	Prejano/ La Rioja		42.19	-2.18	22	21	95.5
34	Valverde del Camino/ Huelva		37.57	-6.74	97	7	7.2
35	Meco/ Madrid		40.56	-3.30	67	26	0
36	Los Barrios/ Cadiz	Thermomediterranean (*n* = 126)	36.22	-5.55	36	10	27.8
37	Castilblanco de los Arroyos/ Sevilla		37.69	-6.00	35	25	71.4
38	Molina de Segura/ Murcia		38.07	-1.20	15	2	6.7
39	Sant Antoni/ Ibiza		38.94	1.41	6	2	33.3
40	Sant Juan/ Ibiza		39.02	1.49	34	25	64.7
41	Fasnia/ Tenerife	Infra-Macaronesian (*n* = 138)	28.22	-16.43	32	15	46.9
42	Arguineguin/ Gran Canaria		27.76	-15.68	106	65	61.3

*Abbreviation*: *n*, number of dogs examined

The dogs examined were hunting dogs living in kennels, stray dogs living in animal protection shelters and shepherd dogs living on farms. Adult flea counts were conducted as described in the WAAVP guidelines [31]. Each dog was inspected for fleas and combed for 5 min over the whole body with a fine-toothed comb. Captured fleas from each infested dog were transferred to a small plastic tube containing 70% ethanol until processing. Fleas were sexed and identified to species under a binocular magnifier according to taxonomic keys [5]. A description of the entomological methodology has been published elsewhere [4].

*Ctenocephalides felis* was the most frequent and widely distributed flea species (82.8% of the surveyed dogs were infested with this species at 71.4% of the surveyed sites) [4]. The data used here for model construction were prevalences (%) of *C. felis* infestation for each collection site, calculated as the number of infested dogs divided by the number of surveyed dogs. Detailed information of the 42 collection sites and *C. felis* prevalences are provided in Table 2.

### Geographical information system. Environmental and climate variables

A geographical information system (GIS) was developed with Arc-GIS 10.4 software using the coordinate reference system ETRS 1989 L Azimuthal Equal Area-LAEA. Within this GIS, each collection site was assigned a set of environmental and climate variables: bioclimatic zone, normalized difference vegetation index (NDVI), altitude, aspect, slope, land cover, distance to urban settlement (UrS), and rainfall and temperature means recorded over the four seasons. The spatial analyst application (SAA) of the GIS software was used to extract the topographical variables (altitude, slope, aspect, land cover) as described below. Altitude was obtained from a 900 m resolution digital elevation model (DEM) from GTOPO30, provided by the U.S. Geological Survey (EROS Data Center, Sioux Falls, South Dakota, USA). Aspect and slope layers were derived from this DEM using the SAA Surface Tool. The aspect identifies the downslope direction of the maximum rate of change in value from each raster cell to its neighbors. The slope is the gradient, or rate of maximum change from each cell of a raster surface. Land cover values were extracted from the 100 m resolution Corine Land Cover (CLC) 2006 raster map of the European Environment Agency (http://www.eea.europa.eu/data-and-maps/data/clc-2006-raster). These data are organised at 3 hierarchical levels, but only the higher level was used to define the following land use categories: artificial surfaces, agricultural areas and forest and semi natural areas. Through the SAA, land cover values for each site were obtained by corresponding land cover extractions. A distance to UrS layer (250 m resolution raster) was built by calculating the Euclidean distance from each cell to the urban settlement feature layer prepared from the 111 and 112 CLC codes (artificial and urban surfaces).

The index NDVI describes the vegetation visualized through a specific combination of two bands, near-infrared (NIR, which is strongly reflected by vegetation) and visible red light (VRL, which is absorbed by vegetation) according to the equation: NDVI = (NIR – VRL)/ (NIR + VRL). From the Visualization Viewer (GloVis: http://glovis.usgs.gov/) we downloaded both NIR and VRL bands from 36 different scenes of the Landsat 8 Collection: L8 OLI/TIRS data set with 5 min of spatial resolution. We have chosen scenes from June 2015 to January 2016 and with less than 10% cloud cover. The NDVI raster layer was then created with the image analysis toolbar in ArcGis v.10.4.

Average temperature and precipitation data layers were obtained from WorldClim 1.4 (http://www.worldclim.org). These are based on interpolated climate data from weather stations for 1960–1990 with 5 min of resolution [32]. Temperature and precipitation values were those recorded for autumn (September-November), winter (December-February), spring (March-May) and summer (June-August).

### Modelling the distribution of *C. felis*

A statistical approach was used to model the predicted prevalence of *C. felis* in dogs in Spain. Generalized linear models were used to estimate prevalences for the binomial family with a logit link, in which environmental and climate factors were used as explanatory variables. The model building strategy was: first, all factors were analyzed by bivariate analysis using odds ratios (together with 95% confidence interval) and then, starting with all variables showing a *P*-value lower than 0.2 in the bivariate analysis, multivariate backward stepwise regression was conducted. The likelihood ratio test was used to compare nested models. To assess the predictive performance of the model, bootstrapping was performed with a 1000 replicates to predict the prevalence of *C. felis* as the number of infested dogs out of the number of surveyed dogs. R-squared was used to compare observed versus expected values. All statistical analyses were performed using Stata v.14 software (StataCorp LP, College Station, Texas, USA). Moran’s I global index of spatial autocorrelation was also calculated to test the null hypothesis of no global spatial autocorrelation. Significance was set at *P* < 0.05.

Predictive maps for mainland Spain and its islands were drawn using the Raster Calculator of the GIS software through modelling on chartable raster layers. Models were constructed based on distance to UrS (100 m resolution Euclidean distances), land cover (100 m resolution), bioclimatic zone shapefile turned into low resolution raster layers, climate layers (5 min spatial resolution rainfall and temperature) and NDVI (30 m resolution).

### Climate projections

Future climate projections were estimated from seasonal mean temperature and rainfall data expected for Spain in 2050 under the IPPC5 climate projections of the global climate model (GCM) known as GFDL-CM3. The representative concentration pathway chosen was RCP45, which represents a moderate-forcing stabilization scenario. This is the most recent GCM climate projection used in the Fifth Assessment IPCC report. The GCM output was downscaled and calibrated (bias corrected) using WorldClim 1.4 (http://www.worldclim.org/cmip5_10m) as the baseline ‘current’ climate. Maps of future *C. felis* prevalences under the premise of the climate change scenario were modelled by incorporating future climate projections.

## Results

### Prevalence of *C. felis*

Moran’s I test showed no evidence of spatial autocorrelation (*Z* = -0.970, *P* = 0.166), so *C. felis* infestation prevalence was estimated using the data collected from the sampling sites (Table 3). The model obtained for prevalence using generalized linear models revealed an effect on *C. felis* prevalences of land cover, bioclimatic zone, mean summer temperature, mean autumn temperature, mean summer rainfall, distance to UrS and NDVI. The coefficients of the regression and the steps to predict the prevalence are described by the following probability equation:

$$ {\displaystyle \begin{array}{c}\mathrm{Probability}=\frac{1}{1+\exp\ \left(\mathrm{Step}\ 2\right)}\\ {}\mathrm{Step}\ 2=-1\times \mathrm{Step}\ 1\\ {}\mathrm{Step}\ 1=\hbox{-} \left(\mathrm{CLC}\ \mathrm{agricultural}\  \mathrm{areas}\times 0.8323046\right)\hbox{-} \left(\mathrm{CLC}\ \mathrm{forest}\ \mathrm{and}\ \mathrm{s}\mathrm{eminatural}\  \mathrm{areas}\times 2.81585\right)\ \\ {}-\left(\mathrm{zone}\mathrm{s}\ \mathrm{C}\ \mathrm{and}\ \mathrm{D}\times 1.955472\right)-\left(\mathrm{zone}\ \mathrm{H}\times 2.062617\right)-\left(\mathrm{zone}\ \mathrm{I}\times 0.045719\right)+\left(\mathrm{zone}\ \mathrm{O}\times 1.728136\right)\ \\ {}+\left(\mathrm{Tmean}\  \mathrm{summer}\times 0.390032\right)-\left(\mathrm{Tmean}\  \mathrm{autumm}\times 0.4751731\right)-\left(\mathrm{Prec}.\mathrm{summer}\times 0.5966327\right)\ \\ {}+\left(\mathrm{distance}\  \mathrm{to}\ \mathrm{UrS}\times 0.0137522\right)-\left( NDVI\ 2.290345\right).\end{array}} $$

**Table 3:** Bivariate and multivariate factors related to *C. felis* infestation prevalence in dogs

	Bivariate OR (95% CI)	Adjusted OR (95% CI)
Corine, level 1		
Artificial surfaces	Ref	Ref
Agricultural areas	0.53 (0.38–0.73)***	0.44 (0.24–0.79)**
Forest and semi natural areas	0.12 (0.07–0.19)***	0.06 (0.03–0.13)***
Spring rainfall	0.76 (0.66–0.87)***	–
Summer rainfall	0.65 (0.52–0.8)***	0.55 (0.36–0.83)**
Autumm rainfall	0.77 (0.68–0.88)***	–
Winter rainfall	0.89 (0.82–0.98)*	–
Spring rainfall	0.76 (0.66–0.87)***	–
Summer rainfall	1.17 (1.1–1.25)***	1.48 (1.27–1.72)***
Autumm temperature	1.15 (1.11–1.2)***	0.62 (0.51–0.75)***
Winter temperature	0.89 (0.82–0.98)*	–
Bioclimatic belt		
Bioclimatic zone G	Ref	Ref
Bioclimatic zones C, D	1.03 (0.63–1.66)	0.14 (0.05–0.36)***
Bioclimatic zone H	0.93 (0.62–1.38)	0.13 (0.05–0.31)***
Bioclimatic zone I	2.25 (1.38–3.68)***	0.96 (0.35–2.64)
Bioclimatic zone O	3.42 (2.11–5.53)***	5.63 (1.50–21.14)*
Elevation in km	0.49 (0.29–0.81)**	–
Aspect	0.83 (0.73–0.94)**	–
Slope	1.02 (0.96–1.09)	–
Distance to UrS	0.99 (0.99–1)	1.01 (1.00–1.02)***
NDVI	47.24 (13.12–170.10)***	0.10 (0.01–1.15)

*Abbreviations*: *OR* odds ratio, *CI* confidence interval, *Ref* Reference category, *UrS* urban settlement, *NDVI* normalized difference vegetation index

*P < 0.05, **P < 0.01, ***P < 0.001

These results indicate the factors Inframacaronesic zone, mean summer temperature and distance to UrS were positively correlated with *C. felis* infestation prevalence. In contrast, two CLC classes (agricultural areas, forest and seminatural areas), four bioclimatic zones (Temperate oceanic, Temperate hyperoceanic, Mesomediterranean and Thermomediterranean), NDVI, mean autumm temperature and mean summer rainfall were negatively correlated with *C. felis* prevalence.

### Projections of *C. felis* distribution using current climate estimates

**Fig. 1:**
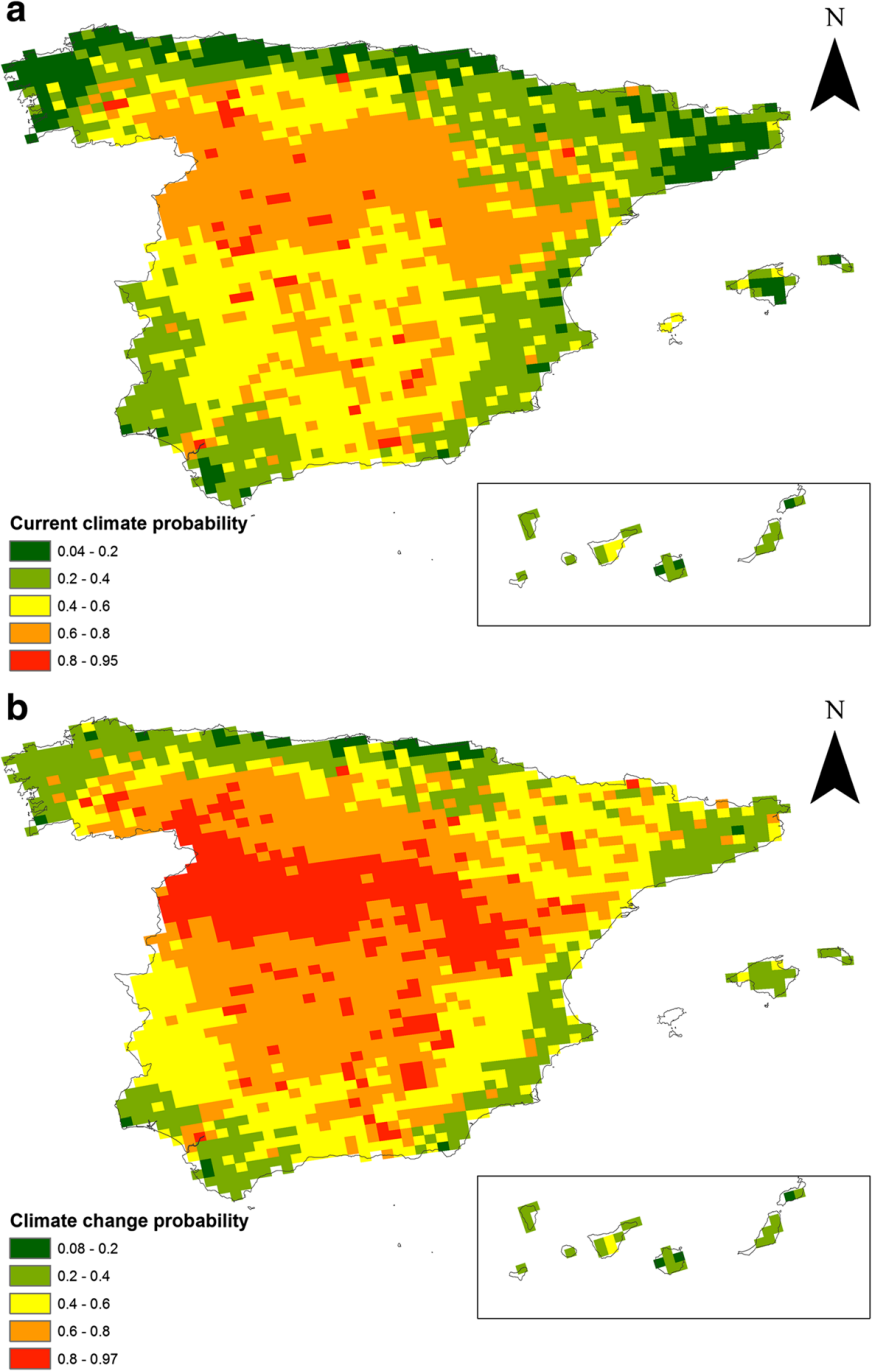
Spatial distributions of the probability of *C. felis* infesting dogs in Spain based on predictive models for current climate conditions (**a**) and future climate scenarios (**b**)

**Fig. 2:**
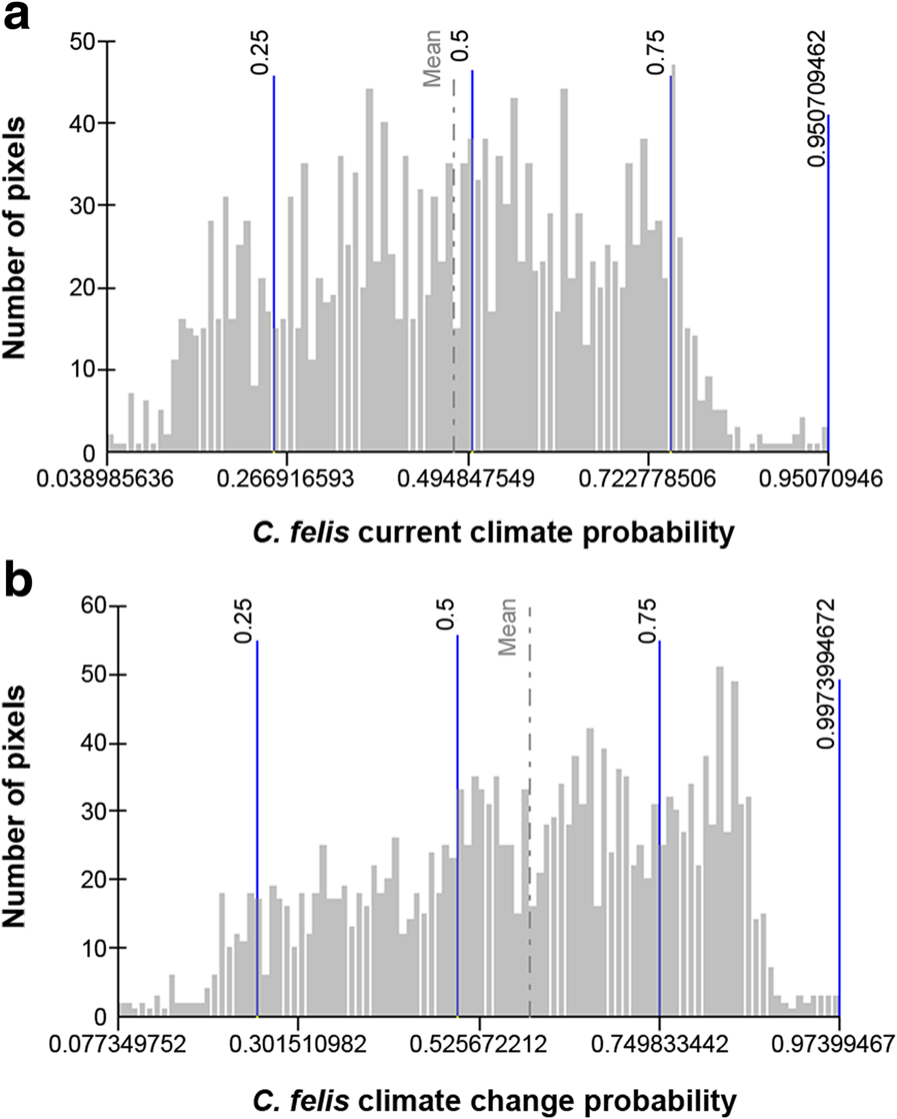
Hystograms of pixel distributions of *C. felis* infestation probability raster models for Spain based on current climate conditions (**a**) and future climate scenarios (**b**)

Based on the prevalence model, 17 km resolution maps were constructed to predict *C. felis* infestation prevalences in Spain using the environmental and climate risk factors identified. The probability was estimated on a scale of 0–1, representing *C. felis* probability of occurrence under the current climate conditions (Fig. 1a). Distributions of pixels in this model approached a multimodal distribution pattern in which several processes showing normal symmetrical distributions are combined (Fig. 2a).

### Projections of *C. felis* distribution using predicted climate change effects

Through simulated climate change expected for Spain in the IPPC5, the predicted spread of *C. felis* prevalences was computed using the GIS software (Fig. 1b). Predicted *C. felis* probability was assumed only on shifts in summer temperature, autumm temperature and summer rainfall. Although other risk factors such as land use will obviously intervene, these are hard to predict with confidence and the complex methods needed are beyond the scope of this study. Figure 1b shows that the risk of spread is slightly greater for the predicted climate change, especially in the north and central part of the country, compared to the current situation. Moreover, the data distribution of pixels in this model shows a left-skewed pattern. The skewed distribution is asymmetrical with a natural limit that prevents outcomes on the right side (Fig. 2b).

## Discussion

In this study, we explored the effects of chartable environmental and climate variables on the spatial distribution of the probability of *C. felis* infesting dogs in Spain, based on predictive models for current climate conditions and future climate scenarios. Some host and habitat variables have been previously identified to affect the flea infestation of dogs in this region [4].

Highest *C. felis* infestation probabilities were detected in the Supramediterranean belt in mainland Spain, corresponding to the central northernmost zone of the mainland part of the country. The Inframacaronesian bioclimatic belt emerged as the most likely zone of *C. felis* infestation out of the six bioclimatic levels analysed. Bioclimatics is an important determinant of habitat suitability for the cat flea and its hosts because it encompasses the effects of both climate and vegetation factors [20, 33]. However, as a limitation for a more in-depth knowledge of a whole bioclimatic area, more sample points are required.

When we considered land cover preferences, the two more natural CLC classes (agricultural areas, forest and seminatural areas) were less correlated with *C. felis* infestation probability than the CLC artificial surfaces. Hence, the cat flea seems to show a preference for more anthropogenic environments which are more populated by reservoir hosts. However, higher *C. felis* infestation probabilities were recorded at sites far from urban areas. Thus, while anthropogenic environments seem to help maintain fleas in peak condition, it could be that densely populated zones are detrimental for these insects.

Higher mean temperatures and lower rainfall in summer increased the likelihood of *C. felis* infestation, while lower autumn temperatures favoured cat flea infestations. When faced with unfavourable climate conditions (too cold, too hot or too dry), flea populations enter a state of diapause as cocoons and wait for these conditions to improve [14]. Owing to the preference shown by the cat flea for the non-green land cover classes and lower rainfall values, a higher NDVI index was negatively correlated with *C. felis* infestation probability. Several studies have positively correlated NDVI with precipitation and observed it is influenced by many factors, especially land cover and climate [19].

The current situation map generated reflects the widespread distribution of the cat flea in Spain, mainly across the central and northernmost belts of mainland Spain. The risk of spread under-predicted conditions of climate changes was found to be slightly higher compared to the current situation, especially in the north and central part of the mainland. The probability of *C. felis* occurrence was scored from 0 to 1, where values close to 0 could indicate diapause rather than absence. As far as we are aware, the literature describes only one climate model (FleaTickRisk) developed to monitor and predict the activity and density of three tick species and the cat flea in Europe, according mainly to temperature and humidity data [14].

Predicting the risk of flea infestation may help in the design of control measures (e.g. to set the frequency of treatments). However, the results provided by our model will need to be adjusted by clinicians to local conditions.

## Conclusions

Under the premise of continued climate change, it is predicted that distributions of fleas and other arthropod vectors will spread because of improved habitat suitability. Data supplied by predictive models, such as those described here, are useful epidemiological tools for veterinarians and other healthcare professionals. Besides improving the advice given to animal owners, these tools serve to design effective programs, based on environment management and the use of insecticides, to control and prevent flea infestations and related diseases caused by flea borne pathogens.

## Abbreviations

CLC: Corine Land Cover
DEM: Digital Elevation Model
ETRS: European Terrestrial Reference System
FAD: Flea allergy dermatitis
GCM: Global Climate Model
GIS: Geographic Information Systems
IPPC: Intergovernmental Panel on Climate Change
LAEA: Lambert Azimuthal Equal Area
NDVI: Normalized Difference Vegetation Index
NIR: Near-infrared
RCP: Representative Concentration Pathway
RS: Remote sensing
SAA: Spatial Analyst Application
UrS: Urban settlement
VRL: Visible red light
WAAVP: World Association for the Advancement of Veterinary Parasitology
